# Thiazolidinones Derived from Dynamic Systemic Resolution of Complex Reversible-Reaction Networks

**DOI:** 10.1002/chem.201304690

**Published:** 2014-02-23

**Authors:** Yan Zhang, Olof Ramström

**Affiliations:** [a] KTH Royal Institute of Technology, Department of ChemistryTeknikringen 30, 10044 Stockholm (Sweden)

**Keywords:** cyclization, dynamic chemistry, enzymes, lipases, resolution

## Abstract

A complex dynamic system based on a network of multiple reversible reactions has been established. The network was applied to a dynamic systemic resolution protocol based on kinetically controlled lipase-catalyzed transformations. This resulted in the formation of cyclized products, where two thiazolidinone compounds were efficiently produced from a range of potential transformations.

Biological systems are generally based on complex networks of chemical reactions, operating under thermodynamic and kinetic control in response to various regulating and evoking factors. The delineation of such networks is of high importance, and constitutes an integral part of the fields of systems biology and chemistry.[1–3] Through the study of the properties and dynamics of such highly complex reactive systems, a better understanding of biological processes can be obtained.

Constitutional dynamic chemistry (CDC) represents in this context a powerful approach, enabling the generation and study of molecular systems. During the last decade, CDC has been adopted in a range of different areas, such as identification of protein ligands, selection of artificial receptors, and development of functional materials.[4–20] The key feature of CDC is its dynamic nature, based on reversible covalent bonds or noncovalent interactions, which enables the involved constituents to mutually interchange under thermodynamic control. The resulting dynamic systems are inherently responsive to internal or external pressures, giving rearrangement of the constituent composition and further amplification of optimally reacting species.

Systems generated using CDC operate under thermodynamic control, generally resulting in the energetically favored equilibrium states. However, the systems can also be coupled to kinetic processes, thereby selecting species in relation to the reaction transition states. This led to the establishment of the concept of dynamic systemic resolution (DSR), in which selective, kinetically controlled routes are coupled to the dynamic systems.[21] Especially enzyme-catalyzed reaction pathways present an efficient methodology,[22–28] applying both catalysis and selectivity on the systems. With this strategy, not only the substrate selectivities of different enzymes could be uncovered, but also the enzyme promiscuities could be challenged, providing useful information for enzymatic synthesis in general.

Recent progress in dynamic chemistry has demonstrated an increasing number of useful reversible reaction types. This development is certainly important in itself, and has also led to an extended application scope. However, the diversities of the designed dynamic systems have still been fairly limited. Therefore, efforts to build dynamic covalent systems with more than one reversible reaction have been explored.[26,28–32]

Although reversible cascade reactions provide easy access to multifunctional structures, parallel reactions generate structures of higher diversity. Thus, by combining these two strategies, an efficient approach to dynamic systems with greatly enhanced complexities can be obtained. Herein, we report a complex dynamic systemic resolution protocol based on networks of multiple reversible reactions, resulting in a range of possible transformations (Figure 1).

**Figure 1:**
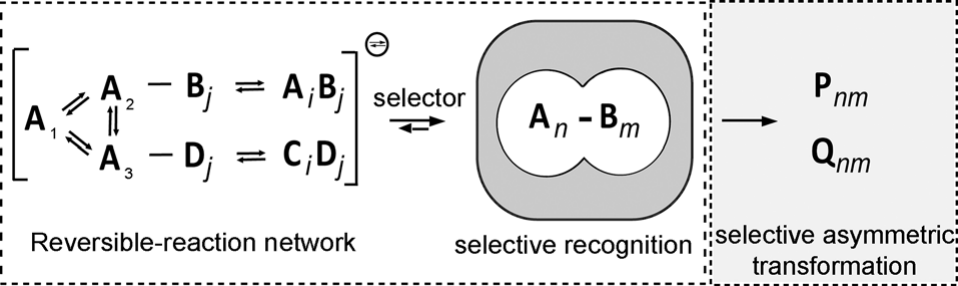
Dynamic systemic resolution of complex reversible-reaction network.

Of the relevant reversible processes available for the reaction network, two were initially chosen: the nitroaldol and hemithioacetal reactions. Although these are able to generate two major types of intermediates for the simultaneous enzymatic transformations,[28] additional diversity was established from the incorporation of imines into the existing system. In principle, this would result in eight main types of reversible reactions operating concertedly: hemiaminal, hemithioaminal, hemithioacetal, aminal, and hydrate formation; and nitroaldol- and aza-nitroaldol reactions (Scheme 1). Due to the dynamic nature of these reactions, all individual components and constituents would mutually undergo interchange under the same conditions. This enhanced reaction complexity would thus result in a large amount of substrates of increased variety, subsequently available for enzyme selection and transformation. Starting from *n* imines, *m* amines, H_2_O, *p* thiols and *q* nitroalkanes, a reaction network composed of (*m*^2^*n*+2*mn*^2^+2*mnp*+2*mnq*+5*mn*+2*m*+*n*^3^+2*n*^2^*p*+2*n*^2^*q*+5*n*^2^+2*p*+2*nq*+6*n*+2*p*+2*q*+2)/2 entities could in principle be formed, not counting chirality.

**Scheme 1:**
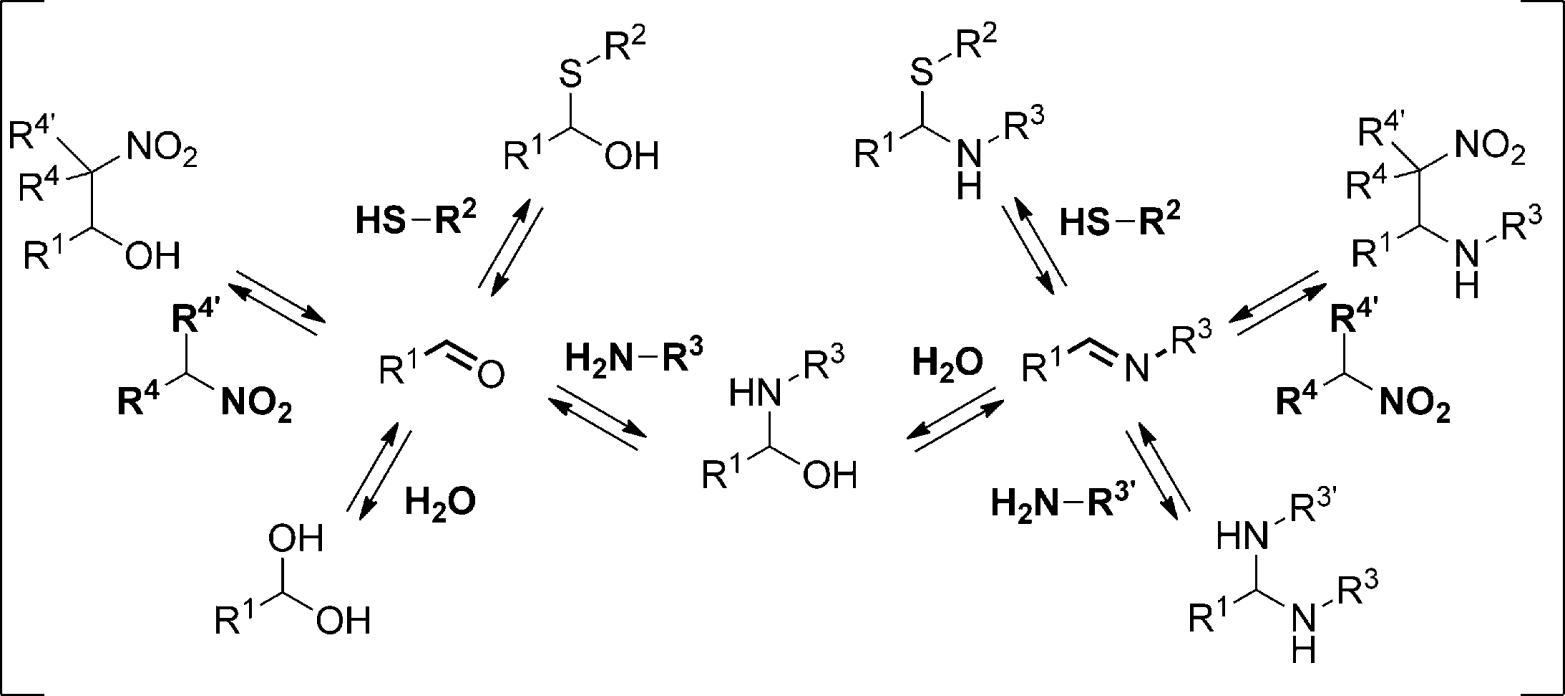
Dynamic covalent reaction network primarily composed of imine formation/hydrolysis, transimination, hemithioacetal/hemithioaminal formation, and nitroaldol reactions.

The simultaneous reversibility of the individual reactions was next addressed, initially investigating the transimination and imine-formation reactions by using a model system composed of 3-nitrobenzaldehyde (**1**), *N*-(3-nitrobenzylidene)methanamine (**2**), and *N*-(3-nitrobenzylidene)propan-2-amine (**3**; Scheme 2).

**Scheme 2:**
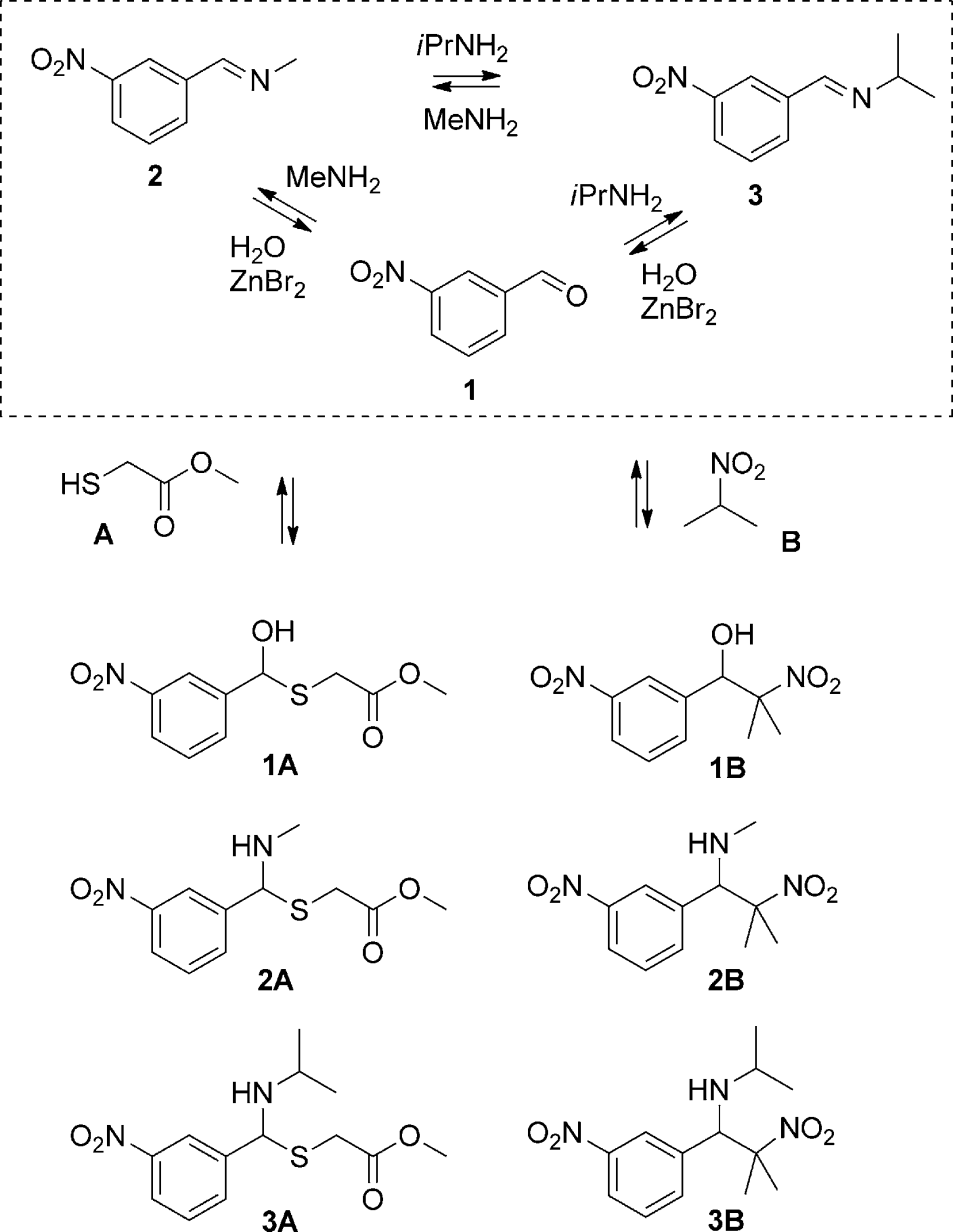
Dynamic model-reaction network.

The transimination between compound **2** and isopropylamine was very fast at room temperature, and equilibrium was reached in one hour. The formation of imine **3** from aldehyde **1** and isopropylamine also went smoothly, with a high conversion of 81 % in one hour. On the other hand, the reverse reaction from imine to aldehyde was slow, and therefore, different additives were tested to accelerate the process. As was expected, addition of base (Et_3_N) did not result in faster conversion. Instead, the Lewis acids Sc(OTf)_3_ and ZnBr_2_, which have been previously proven to be efficient for transimination,[26,33] were tested also in this case. Of these, ZnBr_2_ showed better effects on the reaction rate, and increasing amounts of ZnBr_2_ resulted in faster formation of aldehyde. However, compared to the reaction rate of the imine formation, the reverse reaction was still considerably slower, resulting in a biased distribution between aldehyde and imine. Thus, H_2_O was added to the system to further displace the equilibrium to the aldehyde side. Due to sensitivity of lipases towards water in organic solvents, only two equivalents of H_2_O were added, giving a ratio of 5:1 between imine **2** and aldehyde **1** in 21 h.

Similar to the previous report,[28] the nitroaldol and hemithioacetal reactions showed good reversibility rates and compatibilities when conducted in the same system. Thus, the simultaneous reversible reactions between aldehyde **1**, thiol **A**, and nitropropane **B** could be performed without complications (Scheme 2). Methyl 2-sulfanylacetate **A** was chosen in this case instead of other possible thiols, because the generated intermediates **1 A**, **2 A**, and **3 A** provided additional diversity for the enzyme-catalyzed transformation step: both intermolecular, linear acylations, and intramolecular cyclizations. Replacing the aldehydes by imines in the simultaneous addition reactions proved to be equally efficient. Although for the imine building blocks **2** and **3**, the thiol addition product was relatively unfavored with an equilibrium distribution to the starting material side, the exchange could be recorded by using higher concentrations (20 equiv) of two different thiols. Thus, after addition of the second thiol, immediate redistribution of the constituents took place, confirming fast reversibility rates at room temperature. However, formation of compounds **2 B** and **3 B** was very unfavored, and could not be observed even upon addition of higher amounts of base, and only in large amounts of nitroalkane. Thus, other bases besides Et_3_N were also evaluated, including 4-dimethylaminopryidine (DMAP), and 1,1,3,3-tetramethylguanidine (TMG). Of these, DMAP proved similar to Et_3_N, whereas the stronger base TMG gave small conversions to the adducts. In the latter case, however, potential degradation of the compounds was observed.

Based on the collected properties of the individual reversible reactions, a larger dynamic system was generated with two aromatic aldehydes (**4**–**5**) and four related imines (**6**–**9**), leading to a system size composed of 35 (53 counting chirality) potential species (Scheme 3). Due to the nature of the imines, this represents a subset of the maximal number of potential species. 2-Chloro- and 4-chloro-substituents on the aromatic groups were chosen in this case because of their similar activation effects to the structures. To build the first dynamic system of building blocks **4**–**9**, one equivalent of each aldehyde **5** and imine **6** were added into the system, together with one equivalent of isopropylamine to provide imines **8** and **9**. In addition, two equivalents of H_2_O were included to enable formation of aldehydes, as well as half an equivalent each of Et_3_N and ZnBr_2_. By using ^1^H NMR spectroscopy as a tool to monitor the reaction process, it was again obvious that imine formation and transimination were much faster than the aldehyde formation. However, the distribution of all formed constituents at equilibrium was still suitable for the systemic resolution process (Figure 2). Upon subsequent addition of 2-sulfanylacetate **A** and nitropropane **B**, formation of the nitroalkanols **4 B** and **5 B** was observed in the NMR spectra, whereas the remaining reversible reaction products of the complex dynamic system showed a virtual dynamic character and were not visibly expressed.

**Scheme 3:**
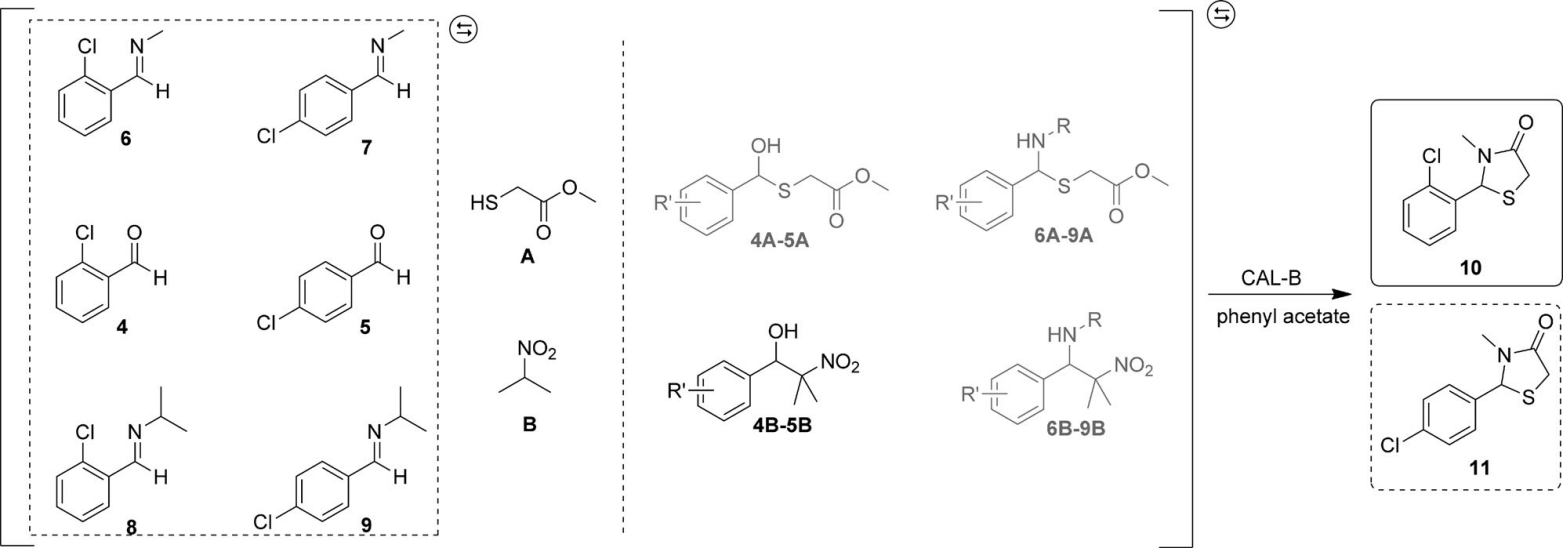
Lipase-catalyzed dynamic systemic resolution of complex reversible-reaction network. Shaded compounds were of virtual nature, not visible in the spectra under the conditions (4 A–5 A, 6 A–9 A, 6 B–9 B). Only compounds 10 and 11 were formed, out of 18 possible kinetic transformations: intermolecular acylation of intermediates 4 A–9 A, 4 B–9 B; or intramolecular cyclization of compounds 4 A–9 A.

**Figure 2:**
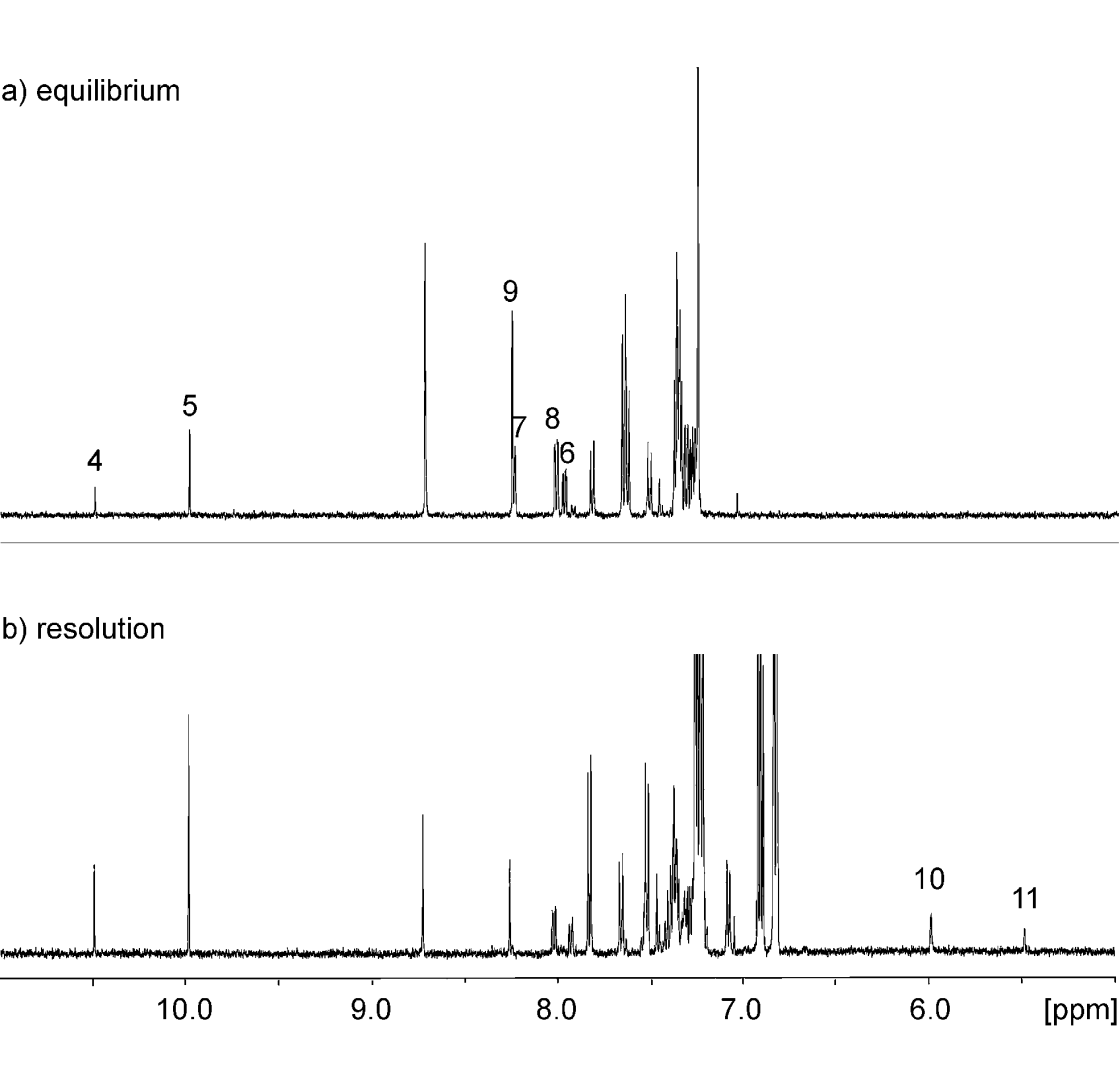
^1^H NMR spectra of complex dynamic multireversible system: a) imine/aldehyde system at equilibrium; and b) overall system upon DSR.

The generated dynamic system was subsequently subjected to further enzyme-catalyzed transformations. Similar to previous studies, lipases were adopted in this case in part due to their many advantages.[34–37] Two different enzyme preparations were thus applied to the complex dynamic system, based on lipases from *Burkholderia* (formerly *Pseudomonas*) *cepacia* (BCL), and *Candida antarctica* (CAL-B), together with phenyl acetate as the acyl donor. A number of possible transformations could in principle be catalyzed by the enzymes in this system, either intermolecular (linear) acylation of intermediates **4 A**–**9 A**, or compounds **4 B**–**9 B**; or intramolecular cyclization of compounds **4 A**–**9 A** (Scheme 3), and the lipase selectivities regarding both substrates and reaction types were next monitored. The transformations were performed at room temperature in *tert*-butyl methyl ether and followed by ^1^H NMR spectroscopy. Conspicuously, only the cyclized products **10** and **11** were observed when applying CAL-B, in spite of the virtual, “invisible”, nature of the parent intermediate structures **6 A** and **7 A** under these conditions. Thus, of the 18 possible kinetic transformations, only two products were formed. These results also represent the first report on this lipase-catalyzed cyclization reaction, showing the potential for lipases to catalyze the formation of thiazolidinone structures. In contrast to CAL-B, BCL provided only marginal amounts of products under the same conditions, likely due to the lower activity compared with CAL-B for these specific substrates. The results can be interpreted by the fact that acylation of hemithioacetal intermediates generally occurs faster than acylation of nitroaldol adducts, and that cyclization is preferred by CAL-B over formation of linear, noncyclized, products. In consequence, only cyclized products were detected from the complex dynamic system. Between intermediates **4 A** and **5 A** and **6 A**–**9 A**, the latter are favored for cyclization due to higher nucleophilicity of the nitrogen atom under these conditions. However, steric factors are likely to also have played a role, because no cyclization of compounds **8 A**–**9 A** could be observed. This result is analogous to previous experiences with lipase-catalyzed amidation, in which larger alkyl groups impaired acylation.[26] The resulting ratio between products **10** and **11** was 2:1, and when considering the equilibrium distribution of intermediates **6** and **7**, compound **10** proved to be the most amplified product from the whole DSR process.

In conclusion, highly complex dynamic systems were generated from networks of different reversible reactions operating simultaneously: primarily imine formation/hydrolysis, transimination, hemithioacetal/hemithioaminal formation, and nitroaldol reactions. By using lipase catalysis with CAL-B to kinetically resolve the complex system, only two compounds were selectively produced out of a large range of potential kinetic products of noncyclized and cyclized nature. In addition, one of the compounds was formed in excess, in response to the enzyme selectivity. This also represents the first report on the formation of thiazolidinones using lipase catalysis. Thus, the compatibility of several different reversible types of chemistry have been demonstrated and successfully coupled to enzyme-catalyzed resolution. The overall DSR process showed high chemoselectivity and amplification capacity. These findings represent an entry into the study and further understanding of complex reaction networks composed of both reversible and irreversible pathways, and can also provide information on biocatalytic activities.
