# Supplementary material for: Thiazolidinones Derived from Dynamic Systemic Resolution of Complex Reversible-Reaction Networks
Source: Chemistry. 2014 Feb 23;20(12):3288–91. doi: 10.1002/chem.201304690 (PMC4497320; doi:10.1002/chem.201304690)

CHEMISTRY
A European Journal

Supporting Information

© Copyright Wiley-VCH Verlag GmbH & Co. KGaA, 69451 Weinheim, 2014

Thiazolidinones Derived from Dynamic Systemic Resolution of Complex Reversible-Reaction Networks

Yan Zhang and Olof Ramström*[a]

chem_201304690_sm_miscellaneous_information.pdf

SUPPORTING INFORMATION

Thiazolidinones From Dynamic Systemic Resolution of Complex Reversible Reaction Networks

*Yan Zhang and Olof Ramström**

KTH Royal Institute of Technology, Department of Chemistry, Teknikringen 30, S-10044 Stockholm, Sweden, ramstrom@kth.se

Table of contents

General methods	S2
Generation of dynamic systems	S2
General procedure for the synthesis of imines as starting materials	S2
Compounds **2-3**, **6-9**	S2
Compound **10**	S3
Compound **11**	S3
NMR spectra of compound **10**	S4
NRM spectra of compound **11**	S4
^{1}H NMR spectra of reversible transimination	S5
^{1}H NMR spectra of imine formation	S6
^{1}H NMR spectra of imine hydrolysis	S6
^{1}H NMR spectra of hemithioaminal formation	S7

General methods

Reagents were obtained from commercial suppliers and used as received. Lipase PS Amano IM (EC 3.1.1.3) was purchased from Amano Enzyme Inc. CAL-B was purchased from Sigma-Aldrich. ^{1}H and ^{13}C NMR data were recorded on a Bruker Avance 400 (100) MHz and/or a Bruker Avance 500 (125) MHz, respectively. Chemical shifts are reported as δ values (ppm) with $CDCl_3$ (^{1}H NMR δ 7.26, ^{13}C NMR δ 77.2) as an internal standard. *J* values are given in Hertz (Hz). Analytical high performance liquid chromatography (HPLC) with chiral stationary phase was performed on an HP-Agilent 1110 Series controller, using a Daicel Chiralpak OJ column (4.6×250 mm, 10 µm). Solvents for HPLC use were of spectrometric grade. Thin layer chromatography (TLC) was performed on precoated Polygram® SIL G/UV_{254} silica plates (0.20 mm, Macherey-Nagel), visualized with UV-detection. Flash column chromatography was performed on silica gel 60, 0.040-0.063 mm (SDS).

Generation of dynamic systems

N-(4-chlorobenzylidene)methanamine (**7**) (0.1 mmol), 2-chlorobenzaldehyde (**4**) (0.1 mmol), isopropylamine (0.1 mmol), H_2O (0.2 mmol), triethylamine (0.05 mmol), $ZnBr_2$ (0.05 mmol), together with dry *tert*-butyl methyl ether (TBME) (0.6 mL) were mixed in a vial, and the resulting mixture stirred at room temperature. ^{1}H NMR was used to follow the reaction process until equilibrium was reached. Then, 2-mercaptoacetate (0.1 mmol), 2-nitropropane (0.1mmol), and phenyl acetate (0.3 mmol) were added into the solution, and the mixture was transferred into a 1.5 mL sealed-cap vial containing CAL-B (5 mg), which had been dried under vacuum for two days. The sealed vials were then kept shaking at room temperature (around 500 rpm), and ^{1}H NMR was used to monitor the reaction progress.

General procedure for the synthesis of imines as starting materials

The corresponding aldehyde (2.4 mmol), amine (4.8 mmol) and $MgSO_4$ (300 mg) were added to a flask together with dichloromethane (7.5 mL). The mixture was stirred at room temperature for 5-7 hours. Then, the $MgSO_4$ was removed by filtration, and the remaining solution evaporated to provide the desired product.

***N*-(3-nitrobenzylidene)methanamine (2)**

Yield: 98%, light yellow solid. ^{1}H NMR (500 MHz, $CDCl_3$, 25 °C) *δ* 3.57 (s, 3H, CH_3), 7.59 (t, *J* = 7.9 Hz, 1H, CH), 8.05 (d, *J* = 7.5 Hz, 1H, CH),8.26 (d, *J* = 7.9 Hz, 1H, CH), 8.35 (s, 1H, CH), 8.54 (s, 1H, CH); ^{13}C NMR (125 MHz, $CDCl_3$, 25 °C) *δ* 48.4, 122.9, 125.1, 129.8, 133.4, 138.1, 148.8, 160.0.

***N*-(3-nitrobenzylidene)propan-2-amine (3)**

Yield: 93%, light yellow solid. ^{1}H NMR (500 MHz, $CDCl_3$, 25 °C) *δ* 1.28 (d, *J* = 6.3 Hz, 6H, $(CH_3)_2$), 3.57-3.65 (m, 1H, CH), 7.58 (t, *J* = 7.9 Hz, 1H, CH), 8.07 (d, *J* = 7.7 Hz, 1H, CH), 8.25 (d, *J* = 7.9 Hz, 1H, CH), 8.37 (s, 1H, CH), 8.56 (s, 1H, CH); ^{13}C NMR (125 MHz, $CDCl_3$, 25 °C) *δ* 24.2, 61.8, 123.0, 124.9, 129.7, 133.7, 138.4, 148.7, 155.8.

***N*-(2-chlorobenzylidene)methanamine (6)**
Yield: 70%, amber liquid. ^{1}H NMR (500 MHz, $CDCl_3$, 25 °C) δ 3.57 (s, 3H, CH_3), 7.26-7.39 (m, 3H, $(CH)_3$), 7.98 (d, J = 7.7 Hz, 1H, CH), 8.73 (s, 1H, CH); ^{13}C NMR (125 MHz, $CDCl_3$, 25 °C) δ 48.6, 127.2, 128.2, 129.9, 131.5, 133.4, 135.0, 159.4.

***N*-(4-chlorobenzylidene)methanamine (7)**
Yield: 90%, white solid. ^{1}H NMR (500 MHz, $CDCl_3$, 25 °C) δ 3.51 (s, 3H, CH_3), 7.38 (d, J = 8.2, 2H, $(CH)_2$), 7.64 (d, J = 8.2 Hz, 2H, $(CH)_2$), 8.24 (s, 1H, CH); ^{13}C NMR (125 MHz, $CDCl_3$, 25 °C) δ 48.3, 129.0, 129.2, 134.8, 136.6, 161.3.

***N*-(2-chlorobenzylidene)propan-2-amine (8)**
Yield: 77%, colorless oil. ^{1}H NMR (500 MHz, $CDCl_3$, 25 °C) δ 1.27 (d, J = 6.4 Hz, 6H, $(CH_3)_2$), 3.57-3.65 (m, 1H, CH), 7.26-7.38 (m, 3H, $(CH)_3$), 8.02 (d, J = 7.7 Hz, 1H, CH), 8.73 (s, 1H, CH); ^{13}C NMR (125 MHz, $CDCl_3$, 25 °C) δ 24.3, 61.9, 127.1, 128.6, 129.8, 131.4, 133.7, 135.0, 155.3.

***N*-(4-chlorobenzylidene)propan-2-amine (9)**
Yield: 92%, light yellow solid. ^{1}H NMR (500 MHz, $CDCl_3$, 25 °C) δ 1.27 (d, J = 6.3 Hz, 6H, $(CH_3)_2$), 3.49-3.58 (m, 1H, CH), 7.37 (d, J = 8.3 Hz, 2H, $(CH)_2$), 7.66 (d, J = 8.3 Hz, 2H, $(CH)_2$), 8.36 (s, 1H, CH); ^{13}C NMR (125 MHz, $CDCl_3$, 25 °C) δ 24.2, 61.8, 128.9, 129.4, 135.1, 136.4, 157.1.

2-(2-chlorophenyl)-3-methylthiazolidin-4-one (10)
Conversion: 71%, colorless oil. ^{1}H NMR (500 MHz, $CDCl_3$, 25 °C) δ 2.83 (s, 3H, CH_3), 3.66 (d, J = 15.4 Hz, 1H, CH_2), 3.76 (d, J = 15.4 Hz, 1H, CH_2), 5.99 (s, 1H, CH), 7.20 (d, J = 7.7 Hz, 1H, CH), 7.27-7.34 (m, 2H, $(CH)_2$), 7.41 (d, J = 7.5 Hz, 1H, CH); ^{13}C NMR (125 MHz, $CDCl_3$, 25 °C) δ 30.6, 32.2, 61.7, 126.7, 127.7, 130.0, 130.4, 132.9, 136.9, 171.8.

2-(4-chlorophenyl)-3-methylthiazolidin-4-one (11)
Conversion: 81%, colorless oil. ^{1}H NMR (500 MHz, $CDCl_3$, 25 °C) δ 2.71 (s, 3H, CH_3), 3.71 (d, J = 15.4 Hz, 1H, CH_2), 3.81 (d, J = 15.4 Hz, 1H, CH_2), 5.48 (s, 1H, CH), 7.25 (d, J = 8.4 Hz, 2H, $(CH)_2$), 7.37 (d, J = 8.4 Hz, 2H, $(CH)_2$); ^{13}C NMR (125 MHz, $CDCl_3$, 25 °C) δ 30.3, 33.1, 64.9, 128.5, 129.5, 135.2, 137.9, 171.3.

NMR spectra for compound 10

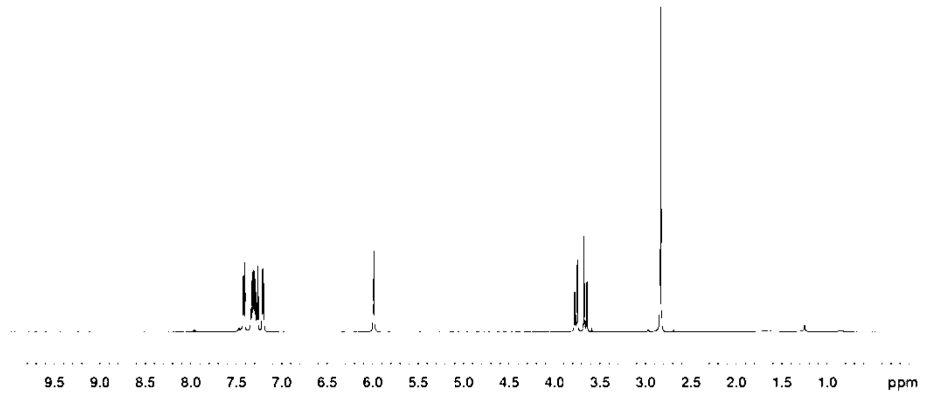

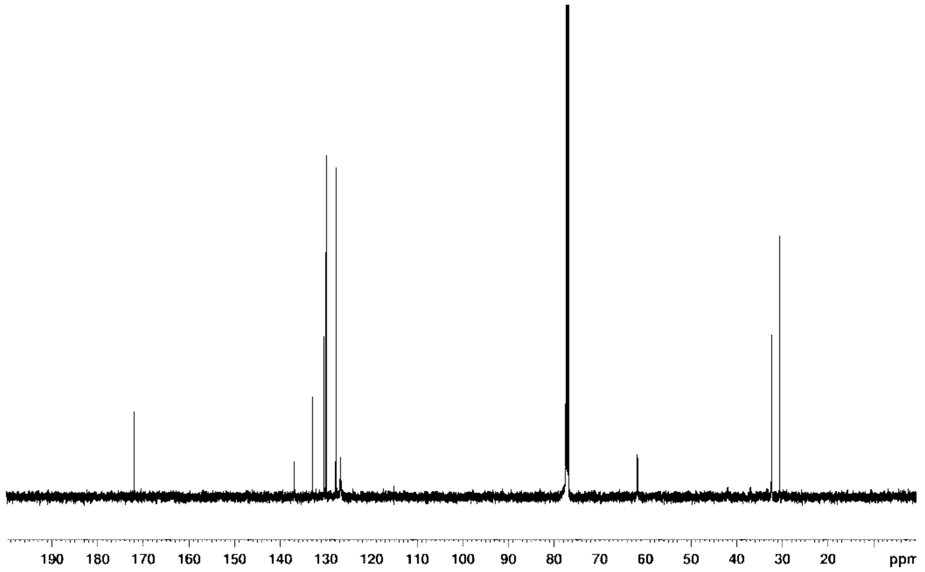

NMR spectra for compound 11

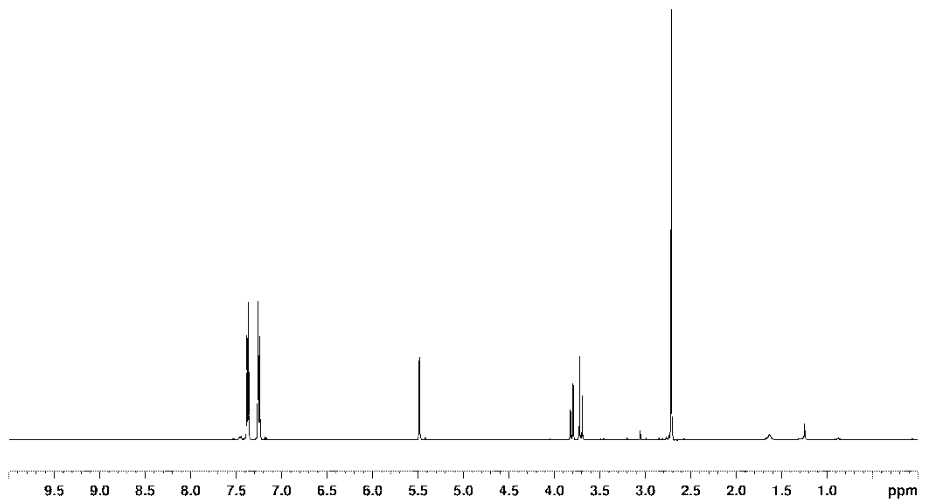

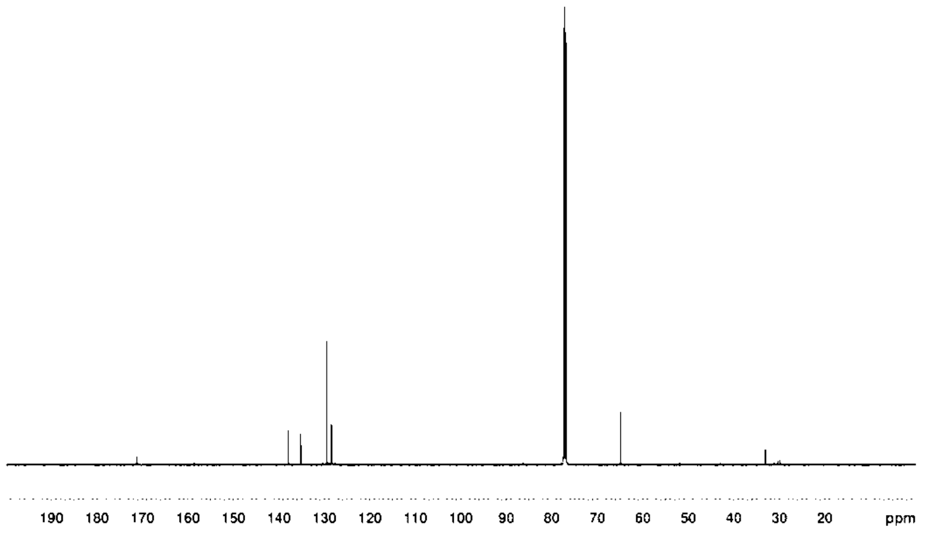

^{1}H NMR spectra of reversible transimination

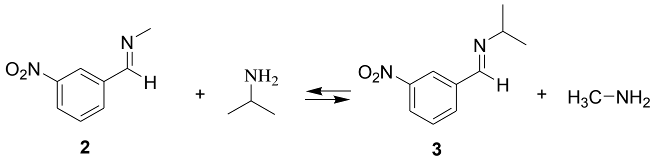

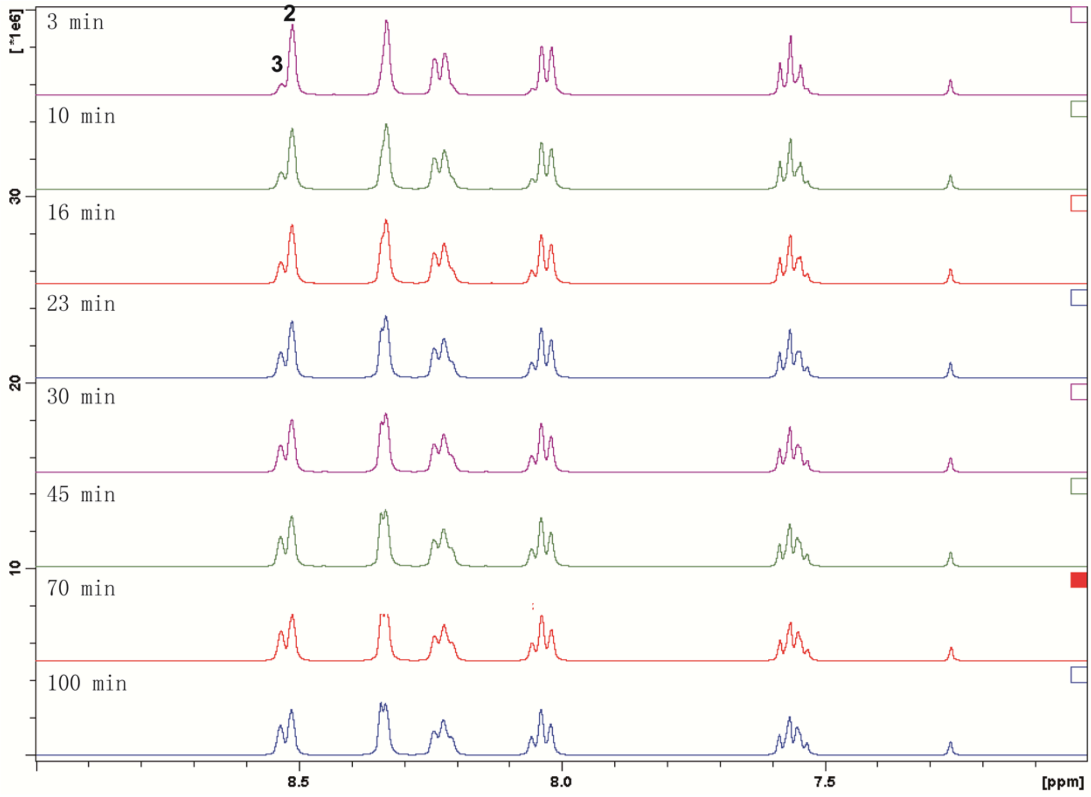

^{1}H NMR spectra of imine formation

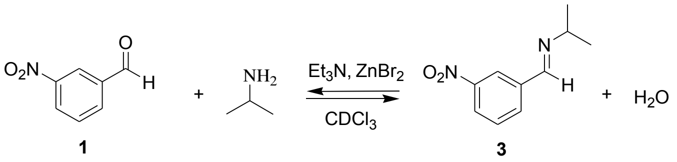

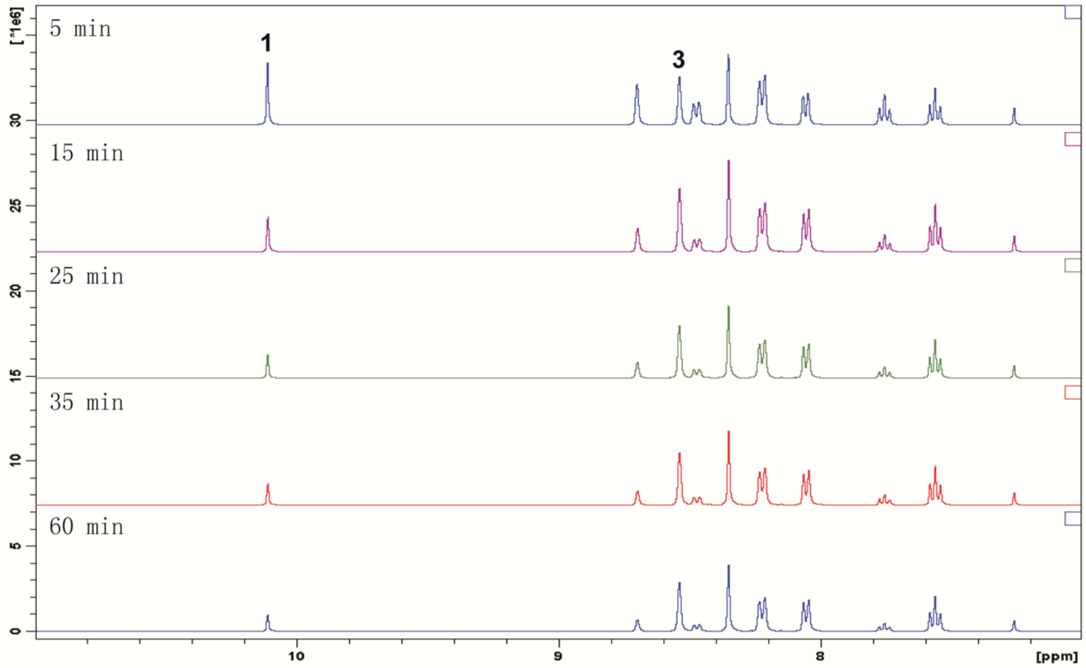

^{1}H NMR spectra of imine hydrolysis

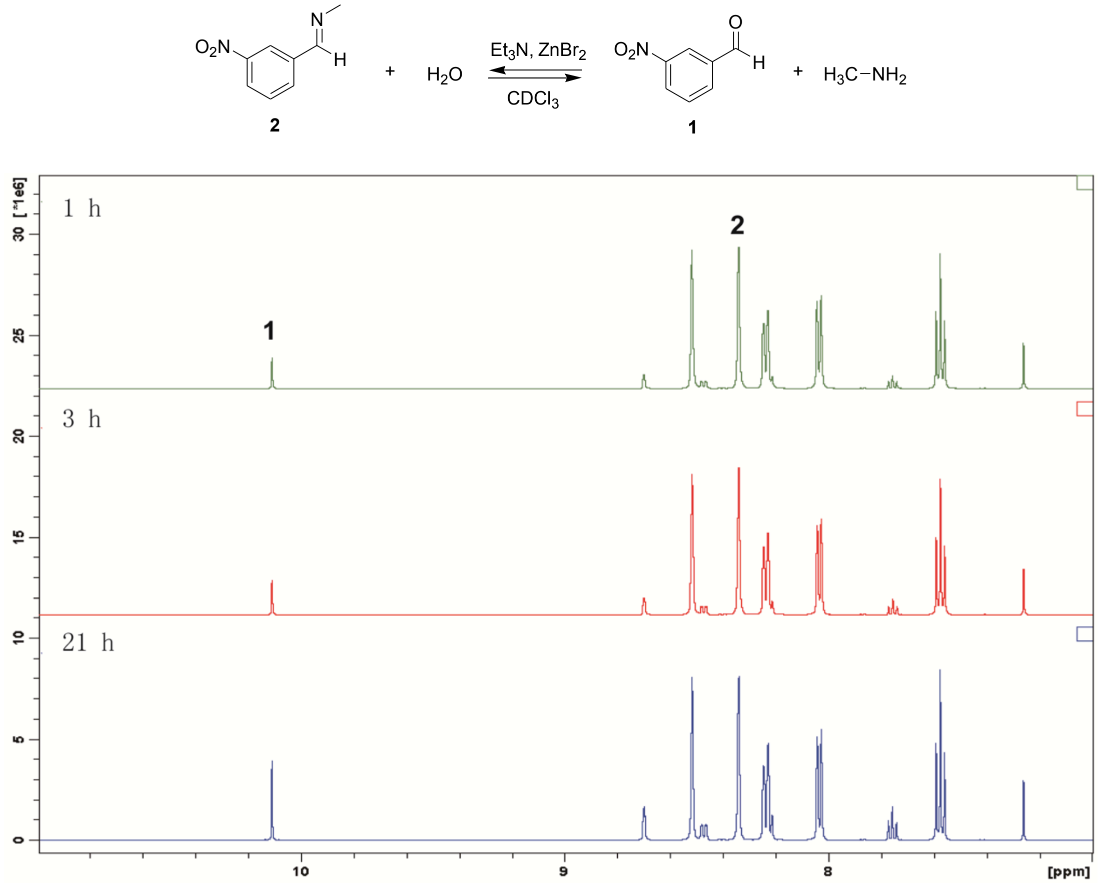

^{1}H NMR spectra of hemithioaminal formation

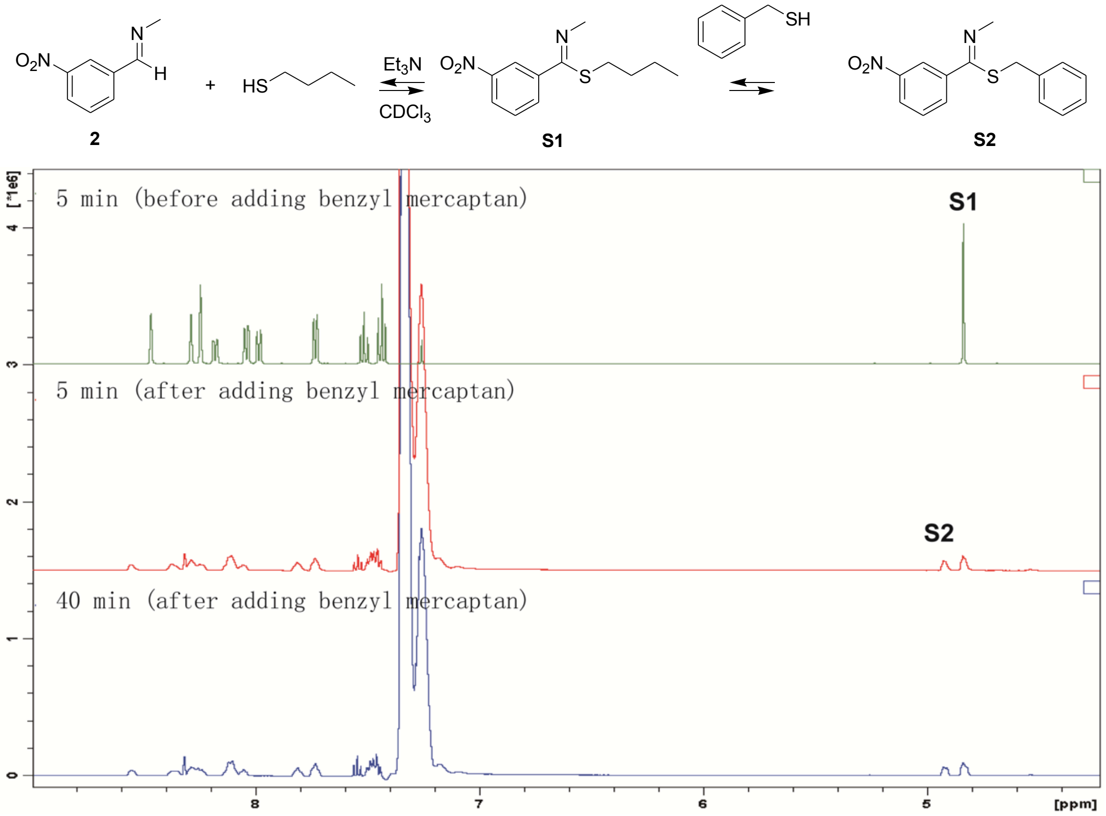

Supplement: Supplementary file 1 [file chem0020-3288-sd1.pdf]
